# Penicillin-binding proteins regulate multiple steps in the polarized cell division process of *Chlamydia*

**DOI:** 10.1038/s41598-020-69397-x

**Published:** 2020-07-28

**Authors:** John V. Cox, Yasser Mohamed Abdelrahman, Scot P. Ouellette

**Affiliations:** 1 Department of Microbiology, Immunology, and Biochemistry, University of Tennessee Health Science Center, Memphis, TN 38163 USA; 2 Department of Pathology and Microbiology, University of Nebraska Medical Center, Omaha, NE 68198 USA; 3 Present Address: Department of Restorative Dentistry, Oregon Health and Science University, Portland, OR 97239 USA; 4 Present Address: Department of Microbiology and Immunology, Faculty of Pharmacy, Cairo University, Cairo, Egypt

**Keywords:** Cell biology, Microbiology

## Abstract

*Chlamydia trachomatis* serovar L2 and *Chlamydia muridarum,* which do not express FtsZ, undergo polarized cell division. During division, peptidoglycan assembles at the pole of dividing *Chlamydia trachomatis* cells where daughter cell formation occurs, and peptidoglycan regulates at least two distinct steps in the polarized division of *Chlamydia trachomatis* and *Chlamydia muridarum*. Cells treated with inhibitors that prevent peptidoglycan synthesis or peptidoglycan crosslinking by penicillin-binding protein 2 (PBP2) are unable to initiate polarized division, while cells treated with inhibitors that prevent peptidoglycan crosslinking by penicillin-binding protein 3 (PBP3/FtsI) initiate polarized division, but the process arrests at an early stage of daughter cell growth. Consistent with their distinct roles in polarized division, peptidoglycan organization is different in cells treated with PBP2 and PBP3-specific inhibitors. Our analyses indicate that the sequential action of PBP2 and PBP3 drives changes in peptidoglycan organization that are essential for the polarized division of these obligate intracellular bacteria. Furthermore, the roles we have characterized for PBP2 and PBP3 in regulating specific steps in chlamydial cell division have not been described in other bacteria.

## Introduction

*Chlamydia trachomatis* is an obligate intracellular coccoid bacterium that is the leading cause of bacterial sexually transmitted infections. This organism undergoes a biphasic developmental cycle during the course of infection. The infectious elementary body (EB) is internalized by target cells and differentiates into the replicative but non-infectious reticulate body (RB) in a membrane-bound intracellular compartment termed the inclusion^1–3^. RBs replicate within the inclusion before secondary differentiation into EBs, which are released from the cell to initiate another round of infection^4^. Although the vast majority of bacteria divide by a highly conserved process termed binary fission that requires the bacterial homologue of tubulin, FtsZ, we recently showed that *Chlamydia trachomatis*, which lacks FtsZ, replicates by a polarized cell division process^5^. This novel polarized mechanism of division is characterized by: (1) the enlargement of a polarized RB, (2) the asymmetric expansion of the major outer membrane protein (MOMP)-enriched pole of the RB, resulting in the formation of a nascent budding daughter cell, and (3) the FtsZ-independent separation of the mother and daughter cell. The morphological changes we observed in *C. trachomatis* serovar L2 (*Ct* L2) as it undergoes division are similar to the polarized budding process that occurs in a subset of the Planctomycetes that also lack FtsZ^6–8^.

Our conclusion that *Ct* L2 undergoes polarized division was based on multiple criteria including a confocal microscopic analysis of fixed and stained cells, which revealed a characteristic distribution of the major outer membrane protein (MOMP), heat shock protein 60 (Hsp60), and DNA during the morphological changes that occur during division. At the completion of polarized growth, which we refer to as the two-cell stage, mother and daughter cells are similar in size and have a similar content and distribution of MOMP, Hsp60, and DNA. As the percentage of polarized division intermediates begin to decline following the initial cell division, there was a corresponding increase in the two-cell stage suggesting that the two-cell stage arises as a consequence of polarized division. We also detected polarized division intermediates in EM analyses, and, importantly live cell imaging of cells labeled with fluorescent sphingomyelin and a GFP-tagged cell division protein revealed a polarized cell division process indistinguishable from that observed with fixed cells, eliminating the possibility that our results were a consequence of artefacts due to our fixation schemes^5^.

To determine whether this mode of division is employed throughout the chlamydial developmental cycle and utilized by other *Chlamydia*, we have quantified the characteristics of dividing *Ct* L2 at various stages of its developmental cycle. In addition, we have characterized and quantified the division process of *Chlamydia muridarum*, which causes genital tract disease pathology in mice^9^ and is only approximately 81% identical at the nucleotide level to *Ct* L2^10^. Our data indicate that this novel mechanism of division is not unique to *Ct* L2 as both *Ct* L2 and *C. muridarum* divided in a polarized manner at all developmental stages examined. Furthermore, inhibitor studies have shown a requirement for peptidoglycan (PG) in two distinct steps of this polarized division process. PG synthesis and its crosslinking via PBP2 are necessary to initiate polarized division. In contrast, cells treated with inhibitors that prevent PG crosslinking via PBP3 initiate polarized division but arrest at a very early stage of nascent daughter cell formation. Consistent with the distinct roles of PBP2 and PBP3 in chlamydial cell division, the organization of PG is very different in cells treated with PBP-specific inhibitors.

## Results

### Quantification of the Polarized division process of *C. trachomatis* serovar L2 in fixed and live cells

**Figure 1:**
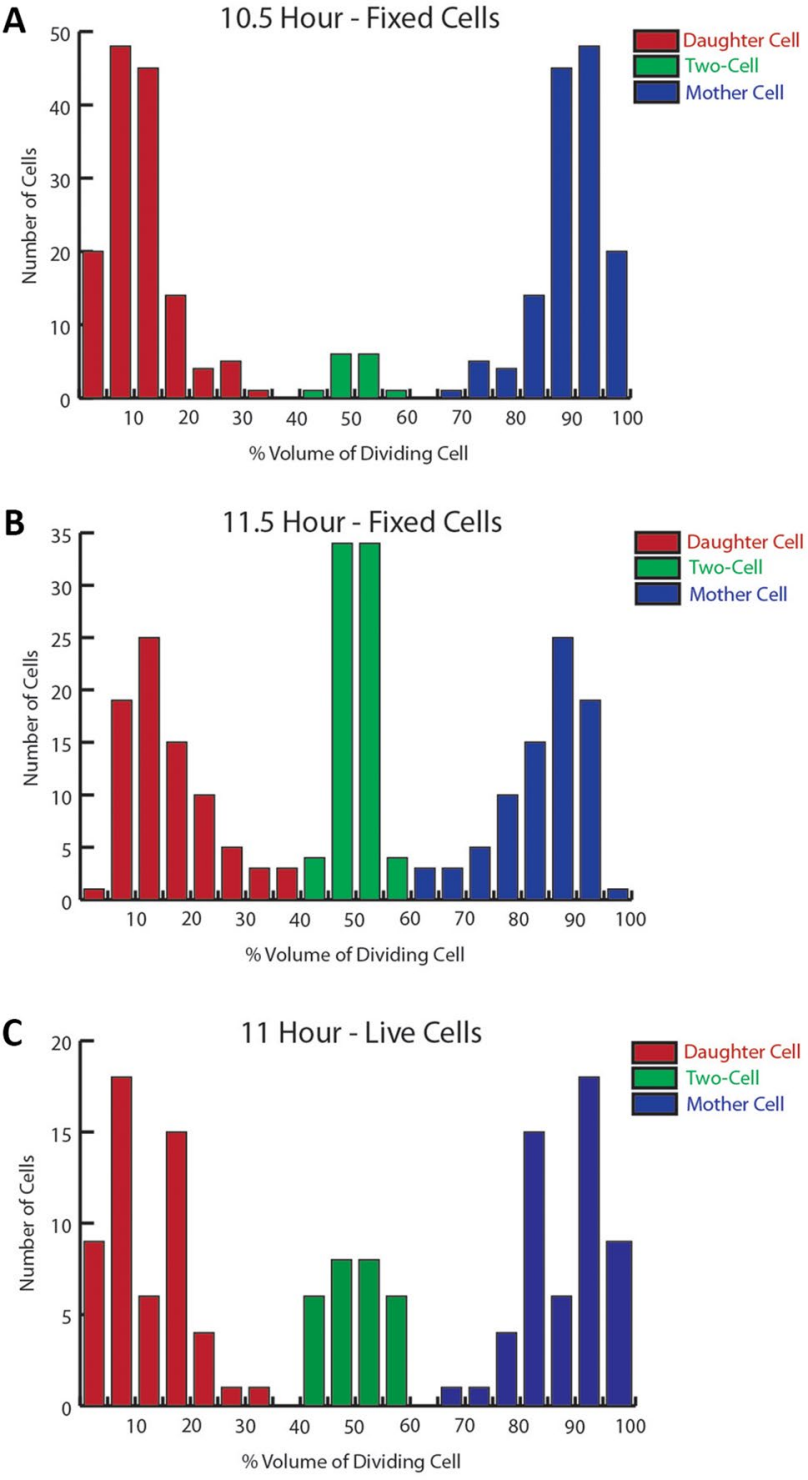
Quantification of daughter and mother cell volume in fixed and live dividing *Ct* L2. HeLa cells infected with *C. trachomatis* serovar L2 were fixed at (**A**) 10.5 or (**B**) 11.5 h post-infection. Following permeabilization, cells were incubated with goat anti-MOMP and rabbit anti-Hsp60 antibodies. The cells were then washed with PBS and incubated with donkey anti-goat IgG conjugated to Alexa Fluor 594 and donkey anti-rabbit IgG conjugated to Alexa Fluor 488. Dividing cells were imaged by collecting Z-stacks that extended above and below the cell on a Zeiss AxioImager. M2 microscope. The largest diameter of the nascent daughter and progenitor mother cell in the dividing RBs was determined using the measurement tool in the Zeiss Axiovision 4.7 software. These values were used to estimate the volume of the daughter and the mother cell. The ratio of the volume of the daughter cell (red) and mother cell (blue) to the total volume of the dividing RB is shown in A (n = 139 cells) and B (n = 112 cells). A subset of the dividing cells has ratios of daughter and mother cells to total RB volumes that fall between 40 and 60%. We designate these dividing cells as the two-cell stage (green). The change in the number of cells at the two-cell stage between the 10.5 and 11.5 h time points is statistically significant (N-1 chi squared: p < 0.0001). The daughter and mother cells in this population are similar in size and have a similar content and distribution of MOMP and Hsp60. (**C**) Alternatively, HeLa cells infected with *C. trachomatis* serovar L2 were incubated with fluorescent BODIPY FL C5 ceramide as previously described^5^. This fluorescent lipid analogue is converted to sphingomyelin and incorporated into chlamydial cell membranes^59–61^. Fluorescently labeled *Chlamydia* undergoing division were imaged in live cells (n = 68 cells) at 11 h post-infection by collecting Z-stacks on a Zeiss LSM710 confocal microscope. These images were used to calculate the ratio of the volume of the daughter cells (red) and progenitor mother cells (blue) to the total volume of the dividing RBs as described in the Materials and Methods. Again, a subset of dividing cells exhibited a ratio of the daughter and mother cell to total RB volume that fell between 40 and 60%. We designate these dividing cells as two-cell stage (green). The images used for the quantification in each panel were acquired from at least two independent experiments. Each dividing cell that was quantified has both a mother and a daughter cell volume represented in the histogram.

To investigate whether a polarized mechanism of division is employed by *Chlamydia* at different stages of its developmental cycle, we quantified the characteristics of dividing *Ct* L2 at various times post-infection. In our initial studies we determined the percentage of the total RB volume the nascent daughter cell and the progenitor mother cell comprise in dividing *Ct* L2 that were fixed and stained with MOMP and Hsp60 antibodies at 10.5 and 11.5 h post-infection (hpi) (Fig. 1). To ensure that the analysis was unbiased, every cell undergoing division was imaged by collecting a Z-stack that extended above and below the dividing cell. The largest diameter of the nascent daughter cell and the progenitor mother cell was determined and used to estimate the total volume of the dividing RB. Representative images from the 10.5 h time point are shown in Supplementary Fig. 1. Since the growth of *Ct* L2 within infected cells is asynchronous, a spectrum of division intermediates was observed at both time points. Consistent with our previous results^5^, polarized division was initiated by an asymmetric expansion of the chlamydial membrane from a pole of the cell that was highly enriched in MOMP giving rise to the nascent daughter cell (hereafter referred to as daughter cell). While MOMP primarily accumulated in the membrane of the growing daughter cell, Hsp60 was almost entirely restricted to the progenitor mother cell (hereafter referred to as mother cell) until late in the division process (Supplementary Fig. 1A). Interestingly, Hsp60 exhibited a reticular pattern of localization throughout the division process (Supplementary Fig. 1A). At 10.5 hpi, the vast majority of dividing cells had daughter cells that comprised a small percentage of the total RB volume (Fig. 1A and Supplementary Fig. 1A). Less than 5% of dividing cells at this time point fell into a category we define as the two-cell stage in which the daughter and mother cells comprise between 40 and 60% of the total RB volume (Fig. 1A). Cells in the two-cell category had a similar content and distribution of MOMP and Hsp60 in the daughter and mother cell (Supplementary Fig. 1A). Identical analyses at 11.5 hpi revealed a small decrease in the population of dividing cells with small daughter cells and a corresponding increase in the two-cell population (Fig. 1B). These data with fixed cell preparations strongly suggest that the two-cell stage, which resembles cells undergoing binary fission, arises as a result of polarized division. We also carried out similar analyses with live *Ct* L2 that were labeled with the fluorescent lipid, BODIPY-sphingomyelin, and imaged at 11 hpi. As previously shown^5^, the polarized cell division intermediates visualized in fixed cells were also detected in live *Ct* L2 labeled with this fluorescent lipid (Supplementary Fig. 1B). In addition, quantification of the daughter and mother cell volume of the dividing cells imaged in this live cell analysis (Fig. 1C) yielded results very similar to those obtained with fixed cells. Taken together, these data support our previous conclusion that the initial division of *Ct* L2 occurs in a polarized manner.

**Figure 2:**
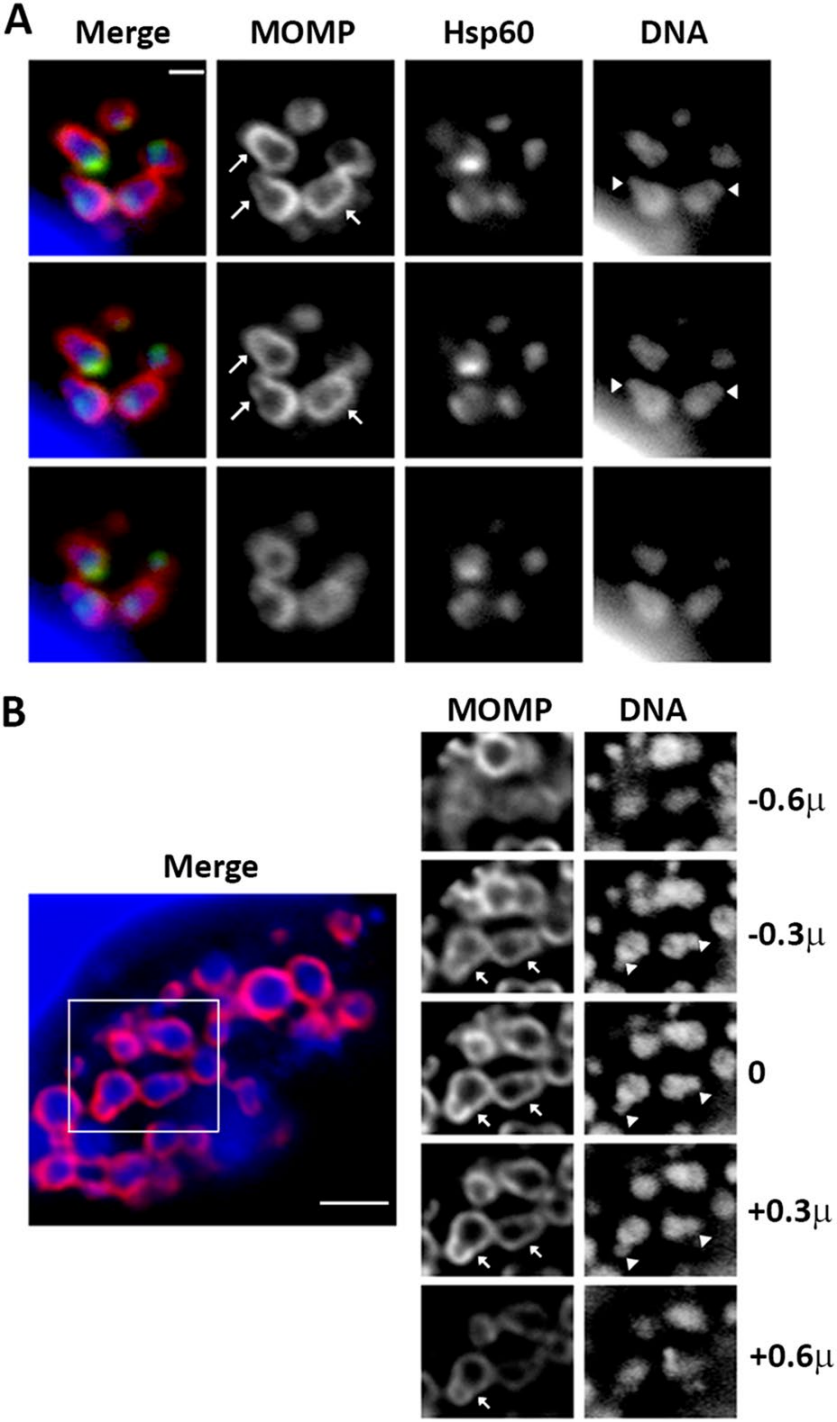
Polarized cell division of *Ct* L2 at later stages of the developmental cycle. HeLa cells infected with *C. trachomatis* serovar L2 were fixed at (**A**) 16 or (**B**) 18 h post-infection. Following permeabilization, cells were incubated with goat anti-MOMP (**A**, **B**) and rabbit anti-Hsp60 (**A**) antibodies. The cells were then washed with PBS and incubated with donkey anti-goat IgG conjugated to Alexa Fluor 594 (red) and donkey anti-rabbit IgG conjugated to Alexa Fluor 488 (green). The cells were also incubated with Hoechst 33342 (blue) to visualize DNA. Cells were imaged by collecting Z-stacks that extended above and below the cell on a Zeiss AxioImager. M2 microscope and images were deconvolved using Zeiss Axiovision 4.7 software. The images in A are consecutive Z-slices (0.3 μM slice size) of an inclusion. The merged color image in (**B**) is a single Z-slice of an entire inclusion, while the individual Z-slices to the right in (**B**) are consecutive Z-slices of the region that is boxed in the merged color image. The numbers to the right in B indicate the Z-plane relative to the Z-slice in the merged color image. Arrows in (**A**) and (**B**) mark cells undergoing polarized division. Arrowheads in (**A**) and (**B**) point to the bacterial chromosome in nascent daughter cells. Note that Hsp60 is almost entirely excluded from nascent daughter cells in (**A**). The white bar in (**A**) is 1 μM and the white bar in (**B**) is 2 μM.

To determine whether *Ct* L2 undergoes polarized division at later stages in its developmental cycle, infected cells were analyzed at 16 and 18 hpi. Images in Fig. 2A,B are representative of the data obtained. At both time points, cells undergoing polarized division could be readily visualized in consecutive sections from a Z-stack in multi-cell inclusions (arrows in Fig. 2A,B). While MOMP was not as polar in dividing cells at these later time points, the accumulation of MOMP in the septum allowed us to determine the boundary between the mother and daughter cell during division. Although a reticular organization of Hsp60 was not observed at these later stages in the developmental cycle, this protein was still primarily restricted to the mother cell until late in the division process (Fig. 2A). Furthermore, the region occupied by the bacterial chromosome was clearly smaller in the budding daughter cells (arrowheads in Fig. 2A,B). Quantification of the daughter and mother cell volume in dividing cells at 18 hpi revealed that a slightly larger percentage of dividing cells fell into the two-cell category at this time point (Supplementary Fig. 2). However, greater than 75% of the dividing cells at 18 hpi had small daughter cells and large mother cells consistent with the notion that *Ct* L2 undergoes polarized division at all stages of its developmental cycle.

### Characterization and quantification of the polarized division process of *C. muridarum*

**Figure 3:**
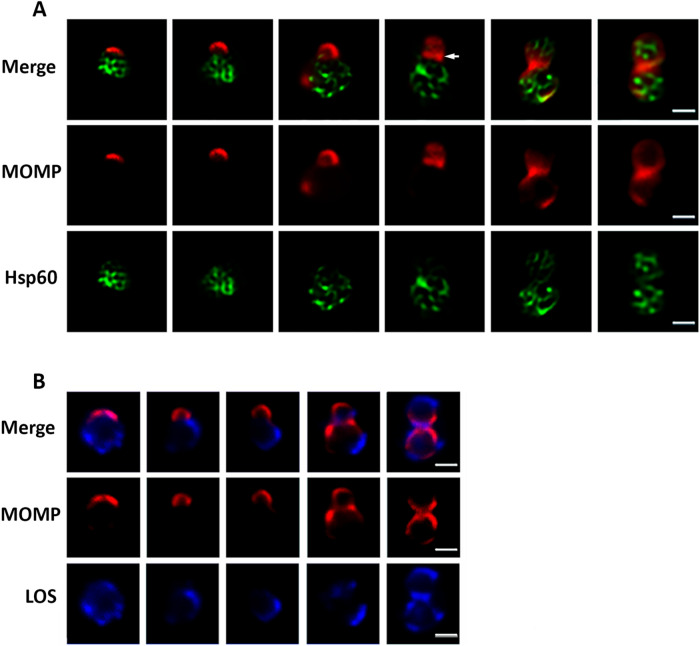
Polarized cell division of *C. muridarum.* HeLa cells infected with *C. muridarum* were fixed at 8 h post-infection. Following permeabilization, the cells were incubated with goat anti-MOMP (red in **A** and **B**), rabbit anti-Hsp60 (green in **A**), and mouse anti-lipo-oligosaccharide (LOS, blue in **B**) antibodies. Cells at different stages of division were imaged by collecting Z-stacks on a Zeiss AxioImager.M2 microscope. Images in A were deconvolved using Zeiss Axiovision 4.7 software. Arrow in A points to MOMP at the septum of a dividing cell. White bars are 1 μM.

**Figure 4:**
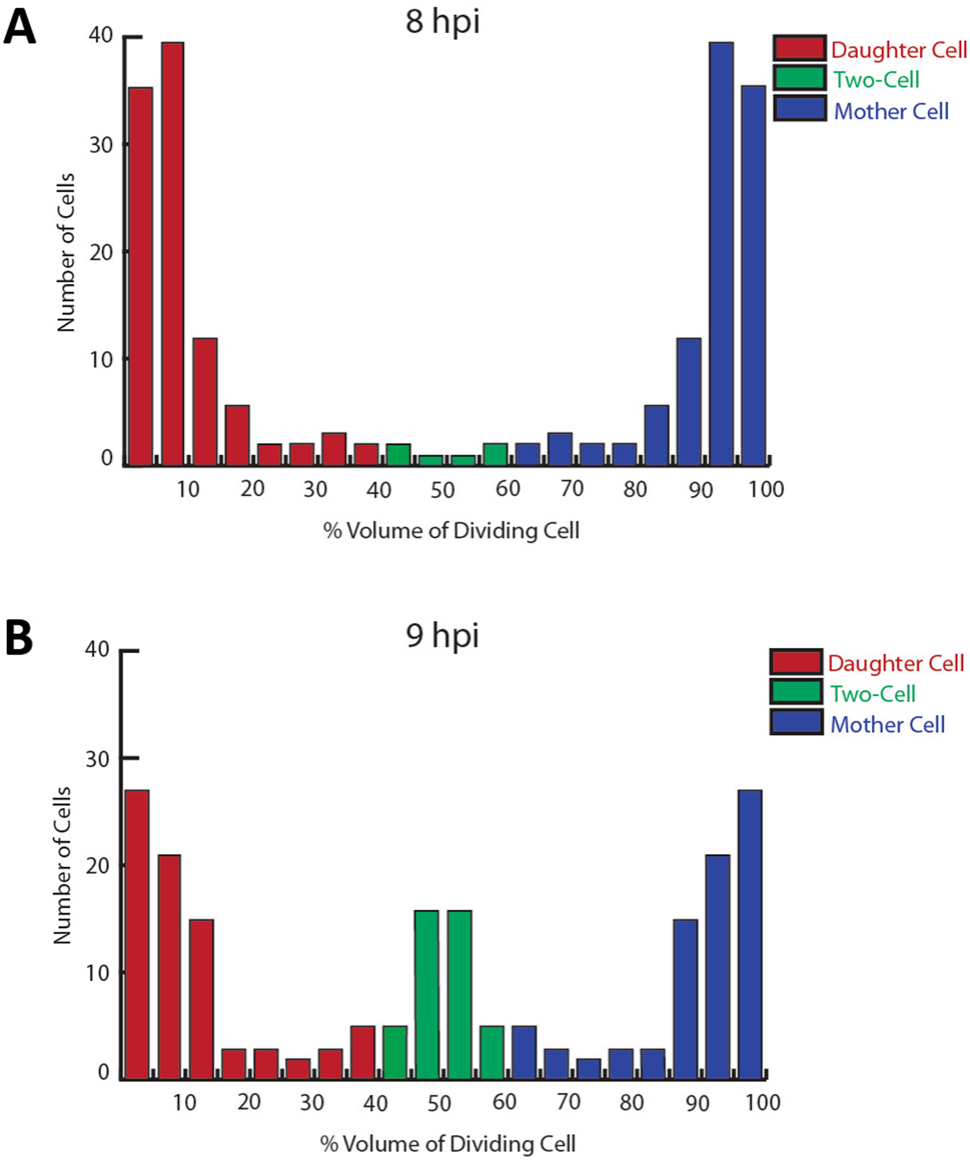
Quantification of daughter and mother cell volume in dividing *C. muridarum*. HeLa cells infected with *C. muridarum* were fixed at (**A**) 8 or (**B**) 9 h post-infection. Following permeabilization, cells were incubated with goat anti-MOMP and rabbit anti-Hsp60 antibodies. The cells were then washed with PBS and incubated with donkey anti-goat IgG conjugated to Alexa Fluor 594 and donkey anti-rabbit IgG conjugated to Alexa Fluor 488. Dividing cells were imaged by collecting Z-stacks that extended above and below the cell on a Zeiss AxioImager. M2 microscope. The largest diameter of the nascent daughter and progenitor mother cell in the dividing RBs was determined using the measurement tool in the Zeiss Axiovision 4.7 software. These values were used to estimate the volume of the daughter and the mother cell. The ratio of the volume of the daughter cell (red) and mother cell (blue) to the total volume of the dividing RB is shown in (**A**) (n = 104 cells) and (**B**) (n = 100 cells). A subset of the dividing cells exhibits ratios of daughter and mother cells to total RB volumes that fall between 40 and 60%. We designate these dividing cells as two-cell stage (green), and the daughter and mother cells in this population are similar in size and have a similar content and distribution of MOMP and Hsp60. The change in the number of cells at the two-cell stage between the 8 and 9 h time points is statistically significant (N-1 chi squared: p = 0.0002). The images used for the quantification in each panel were acquired from at least two independent experiments. Each dividing cell that was quantified has both a mother and a daughter cell volume represented in the histogram.

We also investigated the division process of *Chlamydia muridarum* in infected HeLa cells. This organism causes genital tract disease pathology in mice similar to that caused by the *C. trachomatis* serovars that infect the human genital tract^9^. We found that *C. muridarum* initiates division approximately 8 h after its entry into infected HeLa cells, and divides in a polar manner that is very similar to the polarized division process of *Ct* L2. The first division of *C. muridarum* is initiated by an asymmetric expansion of a MOMP-enriched pole of the cell, which initiates the formation of the daughter cell (Fig. 3). Hsp60, which exhibits a reticular pattern of localization throughout division, is almost entirely excluded from the budding daughter cell until late in the polarized division process (Fig. 3A). In contrast to what was observed in *Ct* L2, MOMP levels decrease at the leading edge of the daughter cell and MOMP accumulates at the septum (arrow in Fig. 3A) prior to the cells reaching the two-cell stage. In addition, lipo-oligosaccharide^11^ is primarily restricted to the mother cell until late in the division process of *C. muridarum* (LOS in Fig. 3B). At the completion of polar growth, the mother and daughter cells of *C. muridarum* are very similar in size and exhibit a similar content and distribution of MOMP, Hsp60, and LOS (Fig. 3). We also quantified the characteristics of dividing *C. muridarum* in infected HeLa cells that were fixed and stained with MOMP and Hsp60 antibodies at 8 and 9 hpi, and the results were very similar to those obtained with *Ct* L2. At 8 hpi when cell division initiates, almost all of the dividing cells had daughter cells that comprised a small percentage of the total RB volume and mother cells that comprised the majority of the dividing RB volume (Fig. 4A). At 9 hpi, there was a small decrease in the population of dividing cells with small daughter and large mother cells and a corresponding slight increase in the two-cell population (Fig. 4B). We conclude from these analyses that the two-cell stage in both *Ct* L2 and *C. muridarum* arises as a consequence of a polarized division process.

### Effect of inhibitors that alter PG synthesis, crosslinking, or stability on the polarized division of *Ct* L2 and *C. muridarum*

Although investigators have been unable to isolate intact peptidoglycan from *Ct* L2*,* mass spectrometry analyses have detected peptidoglycan intermediates or breakdown products in *Ct* L2-infected cells^12^. Furthermore, studies using an ethenyl-modified D-ala-D-ala (EDA-DA) peptidoglycan precursor and Click-Chemistry technology demonstrated that the peptidoglycan in *Ct* L2 accumulates in a ring-like structure at the septum that forms between dividing cells^13–15^, and some of the peptidoglycan ring structures were observed in cells that appear to be undergoing polarized division in multi-cell inclusions^15^. These results provided an explanation for why *Chlamydia* growth can be inhibited with β-lactam antibiotics^16^, which induce the organism to enter an aberrant, persistent growth state^17–21^.

Most coccoid bacteria do not express MreB, the bacterial actin homologue that is required for the synthesis of sidewall peptidoglycan in rod-shaped bacteria^22,23^. However, *Ct* L2 and other members of the *Chlamydiaceae* encode MreB^18,24–26^. We and others have shown that MreB accumulates in continuous^27^ or discontinuous^15^ rings at the septum in *Ct* L2 undergoing division, where it colocalizes with peptidoglycan^15^. The treatment of *Ct* L2 with the MreB inhibitor, A22, disrupts septal peptidoglycan^15,18^ and prevents the replication of *Ct* L2 within infected cells^15,18^.

**Figure 5:**
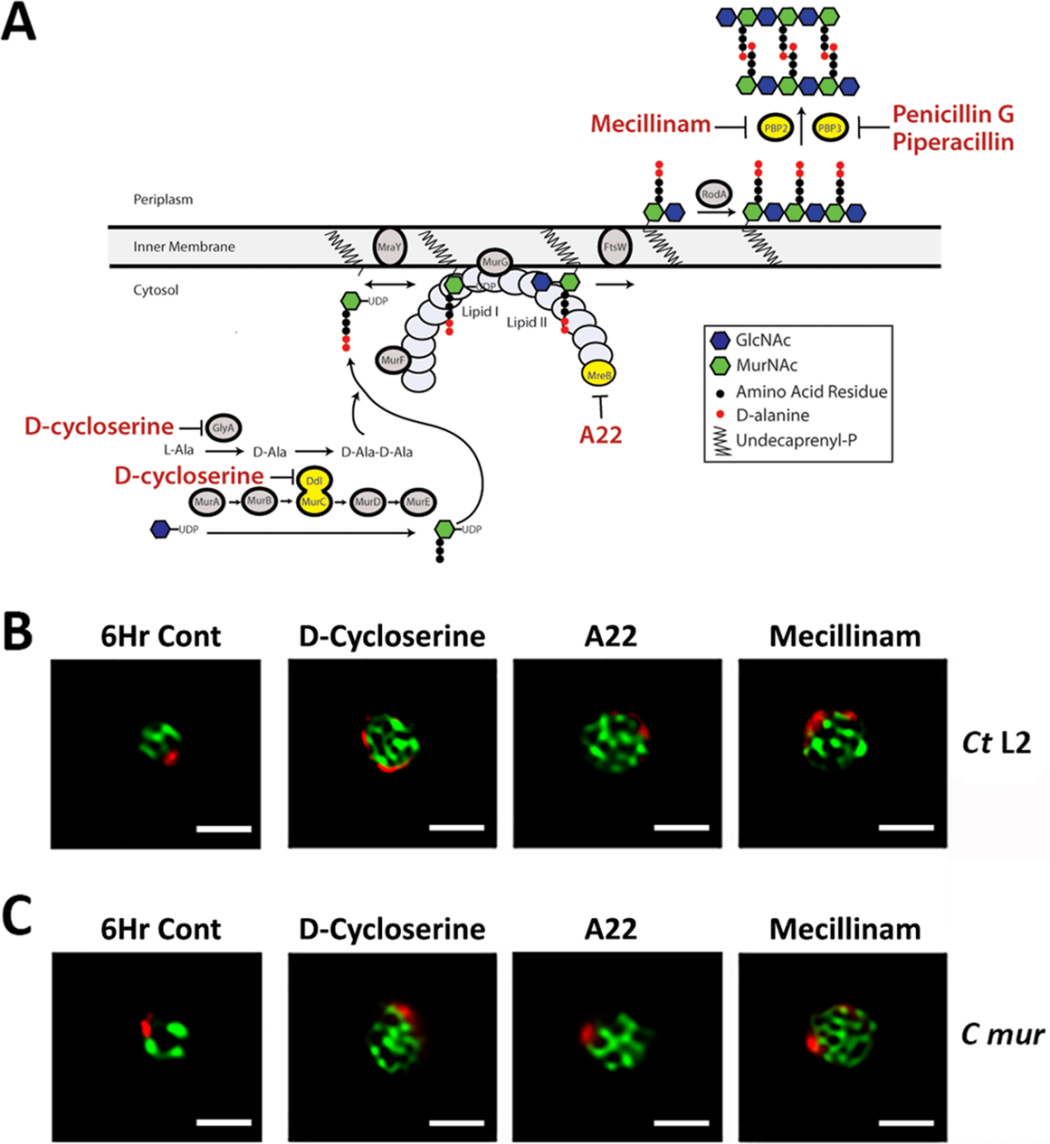
Effect of various inhibitors on the polarized division of *Ct* L2 and *C. muridarum*. (**A**) The peptidoglycan biosynthetic pathway of *Chlamydia* is illustrated. Cartoon was adapted from De Benedetti et al.^62^ and was generated using Adobe Illustrator from Adobe Creative Suite 5 Design Standard. The site of action of the drugs used in our analyses and the role of a subset of biosynthetic enzymes in the pathway are shown. Cartoon depicts hypothetical MreB filament. HeLa cells were infected with (**B**) *Ct* L2 or (**C**) *C. muridarum* (*C mur*). A subset of infected cells was fixed at 6 hpi prior to the initiation of polarized division (6Hr Cont). The fixed cells were permeabilized and incubated with goat anti-MOMP and rabbit anti-Hsp60 antibodies. The cells were then washed with PBS and incubated with donkey anti-goat IgG conjugated to Alexa Fluor 594 (red) and donkey anti-rabbit IgG conjugated to Alexa Fluor 488 (green). Alternatively, 300 μM d-cycloserine, 100 μM A22, or 20 μM mecillinam was added to the infected cells at 6 hpi and the cells were fixed at (**B**) 11.5 hpi or (**C**) 9.5 hpi and stained as described above. Z-stacks of stained cells were acquired on a Zeiss AxioImager.M2 microscope. Images were deconvolved using Zeiss Axiovision 4.7 software. White bars are 1 μM.

To investigate the potential role of peptidoglycan in directing the morphological changes that occur in *Chlamydia* during polarized division, we assessed the effect of various drugs that prevent PG synthesis^28–30^, crosslinking^18,31–33^, or stability^15^ on the division process. For these studies, drugs were added to cells infected with *Ct* L2 or *C. muridarum* prior to the initiation of polarized division at 6 hpi, and the cells were fixed at 11.5 hpi or 9.5 hpi, respectively, and stained with MOMP and Hsp60 antibodies. Cells treated with 300 μM d-cycloserine^14,30^, which prevents the formation of the d-alanine-d-alanine dipeptide that is added to the terminus of the pentapeptide chain of the disaccharide precursor for PG (Fig. 5A), are unable to initiate polarized division. Although *Ct* L2 and *C. muridarum* cells treated with d-cycloserine continue to increase in size following the addition of the drug (Fig. 5B,C), they do not undergo the asymmetric membrane expansion that results in the formation of the daughter cell. A similar result was observed in cells treated with 100 μM A22 (Fig. 5B,C). While d-cycloserine and A22 prevented the asymmetric membrane expansion that occurs at the beginning of polarized division, they had no apparent effect on the reticular organization of Hsp60 in *Ct* L2 (Fig. 5B) or *C. muridarum* (Fig. 5C).

Both *Ct* L2 and *C. muridarum* encode three predicted penicillin-binding proteins that are homologous to *E. coli* PBP2, PBP3, and PBP5/6^24,34,35^. Chlamydial PBP2 and PBP3 are predicted transpeptidases (TPases) that crosslink the pentapeptide sidechains of PG in the periplasm (Fig. 5A), while PBP5/6 has a predicted carboxypeptidase activity^18,24,31^. To investigate the roles of PBP2 and PBP3 in the division process, we treated infected cells with β-lactam antibiotics that preferentially inhibit PBP2 or PBP3. Treatment of infected cells with a concentration of mecillinam (20 μM) that selectively inhibits the TPase activity of *E. coli* PBP2^36^ prevented the asymmetric membrane expansion that occurs at the beginning of polarized division in both *Ct* L2 and *C. muridarum* thus preventing division (Fig. 5B,C). The inhibition of PBP2 with mecillinam affected cell division in a manner very similar to d-cycloserine and A22. This result was unexpected as the inhibition or mutation of PBP2 in other bacteria has primarily been associated with alterations in cell shape, which eventually lead to defects in division^37^. Again, the reticular organization of Hsp60 was not obviously affected by mecillinam treatment (Fig. 5B,C).

**Figure 6:**
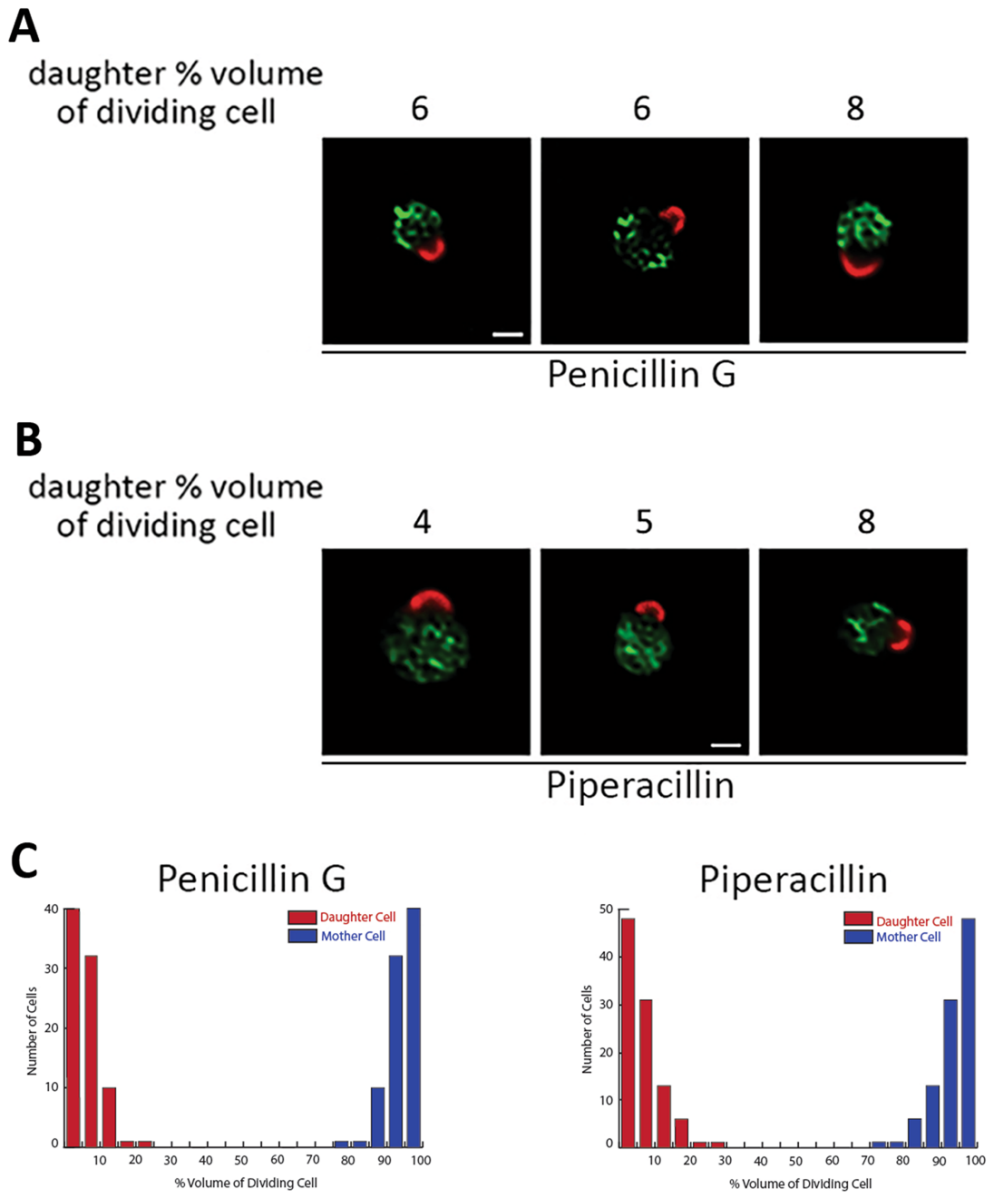
Effect of PBP3 inhibitors on the polarized division of *Ct* L2. HeLa cells were infected with *Ct* L2 and (**A**) 0.2 μM penicillin G or (**B**) 20 μM piperacillin was added to the infected cells at 6 hpi. The cells were fixed at 11.5 hpi and permeabilized. Following washing, cells were incubated with goat anti-MOMP and rabbit anti-Hsp60 antibodies. The cells were then washed with PBS and incubated with donkey anti-goat IgG conjugated to Alexa Fluor 594 (red) and donkey anti-rabbit IgG conjugated to Alexa Fluor 488 (green). The images in panels (**A**) and (**B**), which were deconvolved using Zeiss Axiovision 4.7 software, are representative images illustrating the effect of (**A**) penicillin G and (**B**) piperacillin on division. The number above each cell in A and B is the percent the daughter cell comprises of the total dividing RB volume. In (**C**), the effect of the drugs on the formation of the nascent daughter cell was quantified. Dividing cells were imaged by collecting Z-stacks that extended above and below the cell on a Zeiss AxioImager.M2 microscope. The largest diameter of the nascent daughter and progenitor mother cell in the dividing RBs was determined using the measurement tool in the Zeiss Axiovision 4.7 software. These values were used to estimate the volume of the daughter and the mother cell. The ratio of the volume of the daughter cell (red) and mother cell (blue) to the total volume of the dividing RB for penicillin G-treated cells (n = 104 cells) and piperacillin-treated cells (n = 100 cells) is shown. White bars in A and B are 1 μM.

A different outcome was observed when infected cells were treated with penicillin G, which preferentially inhibits the TPase activity of *E. coli* PBP3^36^ (Fig. 5A). The inhibition of PBP3 or temperature-sensitive mutants of PBP3 alter the crosslinking of septal PG and block cell division resulting in the filamentation of *E. coli*^37^. Penicillin G-treated *Ct* L2 initiated the asymmetric membrane expansion that occurs at the beginning of polarized division, but the cells arrested at a very early stage of nascent daughter cell formation. Representative images are shown in Fig. 6A, and quantification of this analysis revealed that all of the penicillin G-treated cells arrested at a stage in which the nascent daughter cell comprised a very small percentage of the total dividing RB volume (Fig. 6C). Treatment of infected cells with piperacillin, which preferentially inhibits *E. coli* PBP3^36,38^, yielded results very similar to those observed with penicillin G. Piperacillin-treated cells initiated polarized division, but the cells arrested at a stage in which the daughter cell comprised a very small percentage of the total dividing RB volume (Fig. 6B,C).

**Figure 7:**
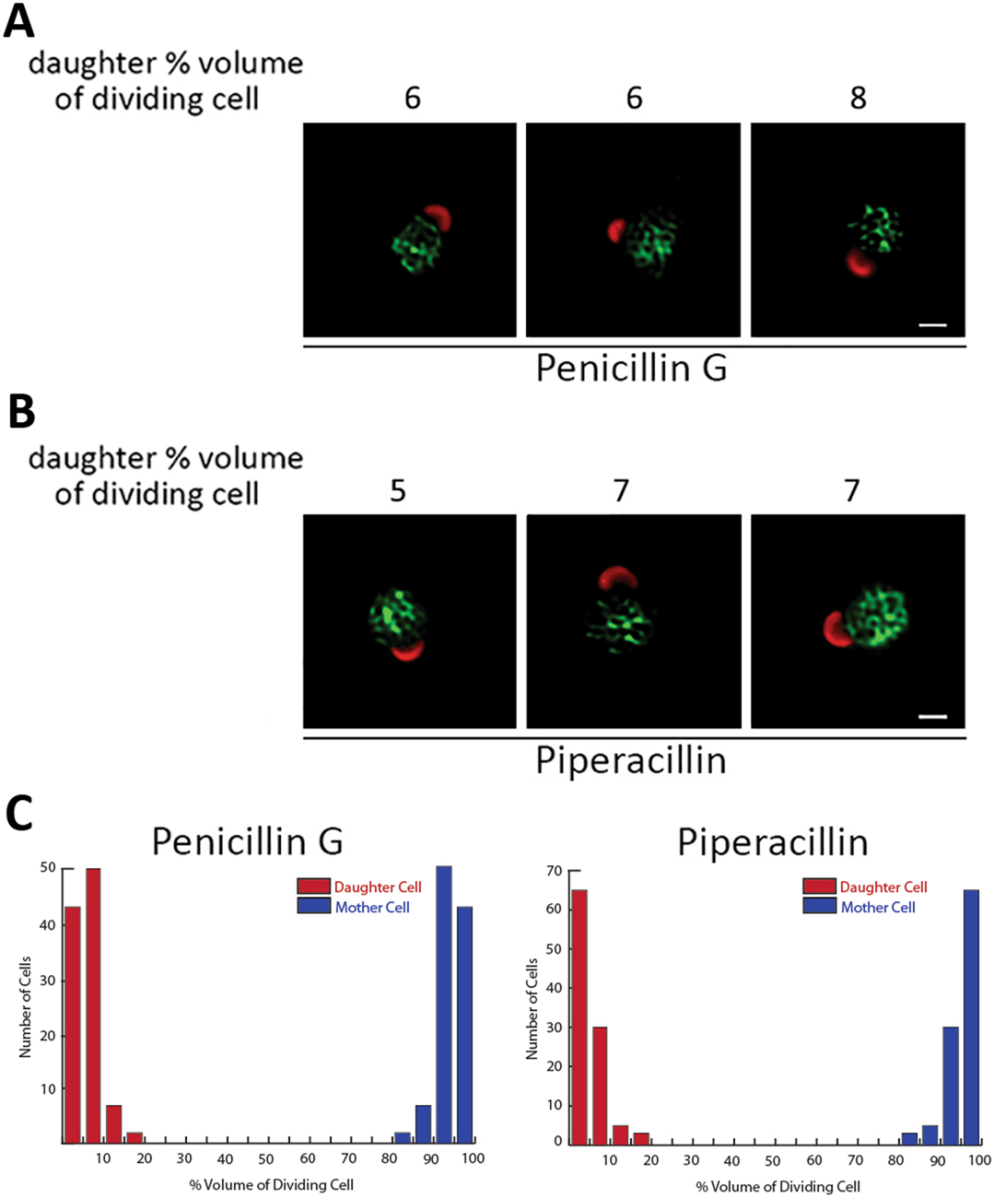
Effect of PBP3 inhibitors on the polarized division of *C. muridarum*. HeLa cells were infected with *C. muridarum* and (**A**) 0.2 μM penicillin G or (**B**) 20 μM piperacillin was added to the infected cells at 6 hpi. The cells were fixed at 9.5 hpi and permeabilized. Following washing, cells were incubated with goat anti-MOMP and rabbit anti-Hsp60 antibodies. The cells were then washed with PBS and incubated with donkey anti-goat IgG conjugated to Alexa Fluor 594 (red) and donkey anti-rabbit IgG conjugated to Alexa Fluor 488 (green). The cells in panels (**A**) and (**B**), which were deconvolved using Zeiss Axiovision 4.7 software, are representative images illustrating the effect of (**A**) penicillin G and (**B**) piperacillin on division. The number above each cell in (**A**) and (**B**) is the percent the daughter cell comprises of the total dividing RB volume. In (**C**), the effect of the drugs on the formation of the nascent daughter cell was quantified. Dividing cells were imaged by collecting Z-stacks that extended above and below the cell on a Zeiss AxioImager.M2 microscope. The largest diameter of the nascent daughter and progenitor mother cell in the dividing RBs was determined using the measurement tool in the Zeiss Axiovision 4.7 software. These values were used to estimate the volume of the daughter and the mother cell. The ratio of the volume of the daughter cell (red) and mother cell (blue) to the total volume of the dividing RB for penicillin G-treated cells (n = 103 cells) and piperacillin-treated cells (n = 103 cells) is shown. White bars in (**A**) and (**B**) are 1 μM.

We have also determined the effect of penicillin G and piperacillin on the polarized division process of *C. muridarum*. In these experiments, inhibitors were again added to infected cells prior to the initiation of division at 6 hpi, and the cells were then fixed at 9.5 hpi and stained with MOMP and Hsp60 antibodies. These analyses yielded results virtually identical to those observed with *Ct* L2. Quantification of these studies indicated that *C. muridarum* treated with penicillin G (Fig. 7A) and piperacillin (Fig. 7B) initiated the asymmetric membrane expansion that occurs at the beginning of polarized division but were arrested at a stage when the daughter cell comprised a very small percentage of the total dividing RB volume (Fig. 7C). These results indicate that PG plays a very similar role in the polarized division process of *Ct* L2 and *C. muridarum.* PG synthesis and its crosslinking via PBP2 is required for the intiation of polarized division in both organisms, while its crosslinking via PBP3 is not required for the initiation of division but is necessary for the continued maturation of the nascent daughter cell and the progression of dividing *Ct* L2 *and C. muridarum* to the two-cell stage.

To determine the effect PBP2 and PBP3 inhibitors have on the intracellular distribution of other cellular markers during division, we have monitored the localization of the translation elongation factor, EF-Tu, and the β subunit of RNA polymerase in *Ct* L2 treated with mecillinam, penicillin G or piperacillin. Our previous studies showed that the β subunit of RNA polymerase was detectable in the daughter cell at an earlier stage in the division process than EF-Tu^5^. Both the polymerase and EF-Tu were uniformly distributed in the cytoplasm of cells that failed to initiate division following mecillinam treatment (Supplementary Fig. 3). Like Hsp60, EF-Tu was retained in the mother cell in cells treated with penicillin G or piperacillin (Supplementary Fig. 3), while the β subunit of RNA polymerase was present both in the mother cell and in the arrested daughter cell following treatment with these antibiotics (Supplementary Fig. 3). These results suggest that preventing PBP3 crosslinking of PG does not prevent the normal partitioning of cytosolic proteins in cells undergoing division. In addition, this imaging analysis revealed that both EF-Tu and the β subunit of RNA polymerase exhibited an organized pattern of localization within cells that may reflect regions of high transcriptional and translational activity.

### Effect of PBP2 and PBP3 inhibitors on PG organization in *Ct *L2 and *C. muridarum*

**Figure 8:**
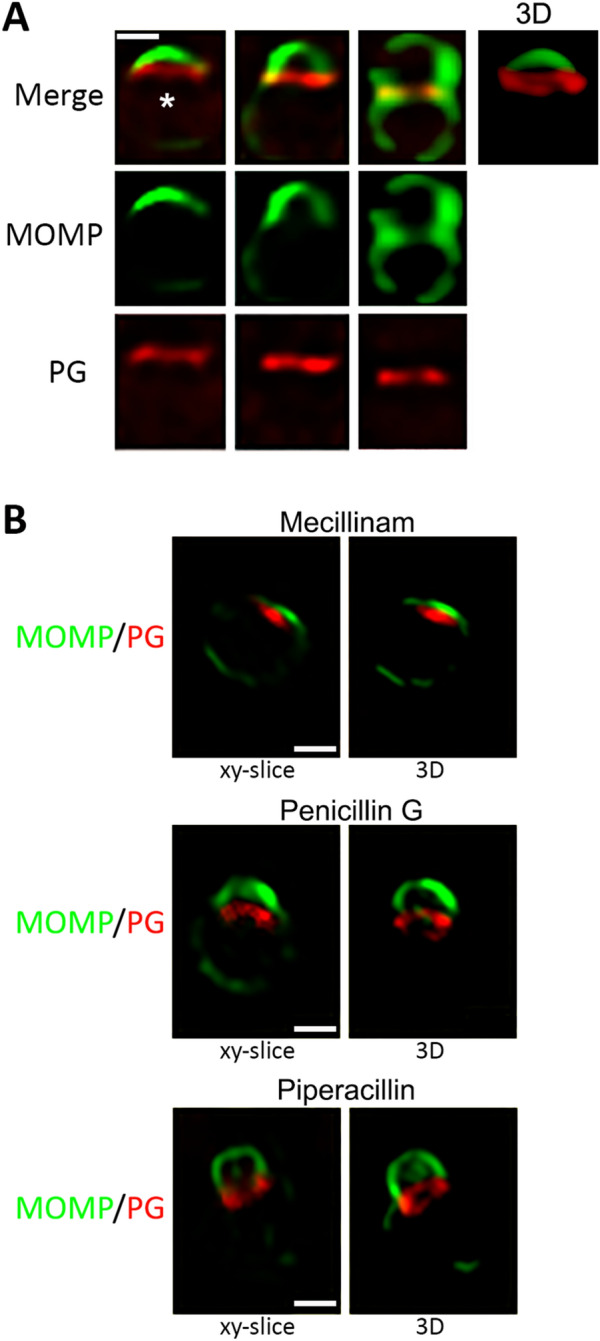
Distribution of peptidoglycan during the first division of *Ct* L2 in the absence and presence of PBP inhibitors. HeLa cells were infected with *Ct* L2 and the peptidoglycan precursor, EDA-DA, was added to infected cells at 7.5 hpi. The cells in panel A were fixed at 10.5 hpi and permeabilized. The cells were rinsed and incubated with goat anti-MOMP antibodies while the EDA-DA was labeled with Alexa Fluor 594 (red) using click chemistry technology (Invitrogen). The cells were rinsed and incubated with donkey anti-goat IgG conjugated to Alexa Fluor 488 (green). Cells at different stages of polarized division were imaged by collecting Z-stacks on a Zeiss LSM710 confocal microscope and the images were deconvolved using Zeiss Axiovision 4.7 software. The panel to the right in A is a 3-dimensional projection of the cell marked with the asterisk that was generated using Zeiss Zen Blue software. (**B**) Alternatively, 20 μM mecillinam, 0.2 μM penicillin G, or 20 μM piperacillin was added to the infected cells at the same time as the EDA-DA. The cells were fixed at 11.5 hpi and processed as described above. Collected images were deconvolved using Zeiss Axiovison 4.7 software. The panels on the left are an xy-slice from the collected Z-stack, and the panels on the right are 3-dimensional projections of the Z-stack generated with Zeiss Zen Blue software. White bars in (**A**) and (**B**) are 1 μM.

Previous studies have primarily examined the distribution of PG in *Ct* L2 that is at very late stages of the polarized division process^14,15^. To assess the distribution of PG in *Ct* L2 at various stages of polarized division we added the PG precursor, ethenyl-d-alanine-d-alanine (EDA-DA), to infected cells at 7.5 hpi. The cells were then fixed at 10.5 hpi, processed for click chemistry to detect the EDA-DA, and stained with antibodies against MOMP. These analyses revealed that PG accumulated at the septum separating the nascent daughter cell from the mother cell very early in the polarized division process and was retained in the septum throughout division (Fig. 8A). A 3-dimensional projection of the cell marked with an asterisk in Fig. 8A illustrates that PG forms a septal ring-like structure at the base of the daughter cell early in division. The exact time when PG synthesis initiates in cells undergoing division is unclear. However, the MOMP-enriched membrane of the nascent daughter cell was always clearly separated from PG in our analyses. Additional studies have examined the distribution of PG in *C. muridarum* as it undergoes cell division. In these assays, EDA-DA was added to infected cells at 6 hpi and the cells were then fixed at 8.5 hpi. Like the results observed with *Ct* L2, PG was initially synthesized just beneath the membrane of the nascent daughter cell very early in the polarized division process of *C. muridarum* and was retained in the septum throughout division (Supplementary Fig. 4A). The 3-dimensional projection of the cell marked with an asterisk in Supplementary Fig. 4A illustrates that PG forms a ring-like structure at the base of the daughter cell early in division.

Similar PG labeling experiments were performed in infected cells treated with the PBP inhibitors described above. In these assays, the drug was added to the infected cells at the same time as the EDA-DA at 7.5 hpi, and labeled cells were fixed at 11.5 hpi. Treatment of cells with mecillinam, which inhibits PG crosslinking by PBP2, prevented asymmetric membrane expansion and PG accumulated in a polar patch just beneath the MOMP-containing outer membrane (Fig. 8B). The 3-dimensional projection of this cell illustrates there is no clear separation of the PG patch from the MOMP-containing outer membrane, and PG rings were not observed in mecillinam-treated cells. A different outcome was observed in cells treated with the PBP3 inhibitors, penicillin G and piperacillin. The xy slices and 3-D projections in Fig. 8B illustrate that partial or complete PG rings form at the base of nascent daughter cells whose growth was arrested as a result of treatment with penicillin G or piperacillin. Additional analyses revealed that mecillinam, penicillin G and piperacillin affect PG organization in *C. muridarum* (Supplementary Fig. 4B) in a manner very similar to that observed in *Ct* L2. These results indicate that the TPase activities of PBP2 and PBP3 have distinct roles in driving changes in PG organization required for the polarized division process of this obligate intracellular bacterium. Since cells treated with these inhibitors arrest prior to the two-cell stage, we have been unable to investigate the potential roles of PBP2 and PBP3 in the step that separates cells at the end-point of division.

## Discussion

Bacteria in the related *Planctomycetes* and *Chlamydiae* phyla lack FtsZ and a subset of these FtsZ-less organisms, which are both free-living and obligate intracellular bacteria, divide by polarized budding^5–8^. The changes in cellular morphology that occur during this polarized division process were previously characterized for *Ct* L2^5^. Here, we have quantified the division intermediates that arise in *Ct* L2 and a related species, *C. muridarum*, through a combination of fixed cell and live cell imaging analyses and 3-dimensional reconstructions. These studies have shown that the initial division of these organisms occurs in a polarized manner. Furthermore, we show that this polarized division process is likely the primary mode of division employed by *Ct* L2 until later stages of its developmental cycle when secondary differentiation has begun.

Our past and current results remain in contrast to a recent report that used 3-dimensional reconstruction of electron microscopy sections to investigate potential mechanisms that control RB to EB differentiation of *Ct* L2. This study concluded that *Ct* L2 divides by binary fission^39^. This conclusion was dependent upon the criteria the authors used to define whether a cell was undergoing division, which relied on identifying a plane of constriction. The early stages of the polarized budding process do not have a traditional constriction site and were likely overlooked or excluded as outliers by the authors. By contrast, our fluorescence assays detect early division intermediates that are easily visualized by imaging the distribution of several cellular markers (MOMP, Hsp60, LOS, PG, and DNA). Indeed, our conclusions are reinforced by the presence of *Ct* L2 undergoing what appears to be polarized division in images presented in this previous 3-dimensional electron microscopic analysis^39^. The similar results obtained in our live cell and fixed cell analyses indicate that polarized division is the primary mode of division employed by *Ct* L2 at all stages of the developmental cycle we examined.

Chlamydiae synthesize PG necessary for cell division in an MreB-dependent manner, and the PG in these organisms is restricted to the septum where it forms a ring. We demonstrate here that PG rings assemble in a polar fashion in *Ct* L2 and *C. muridarum* at an early step in the formation of the daughter cell. This PG ring is initially detected underneath the MOMP-enriched pole of the cell as the nascent daughter cell begins to form. The distance between this PG ring and the leading edge of the growing daughter cell increases as the polarized division process progresses (Fig. 8A). These results further strengthen the model of polarized division in *Chlamydia* by incorporating another key component of the division process, PG, in addition to the markers previously used.

PG synthesis and remodeling require TPases that crosslink the peptide side chains of PG strands. The chlamydial genome encodes two class B monofunctional TPases, PBP2 and PBP3/FtsI^18,34^. PBP2 in rod-shaped bacteria is associated with the MreB-dependent elongasome complex that is required for sidewall PG synthesis^37,40^. Inhibition of *E. coli* PBP2 results in spherical cells that at least initially can continue to divide^37^. PBP3, in contrast, is a component of the FtsZ-dependent divisome complex^41–43^ that accumulates at the septum of dividing cells in both rod-shaped and coccoid bacteria^44–47^. The inhibition of the TPase activity of PBP3 prevents septal PG crosslinking^48,49^ and blocks fission^37,50,51^. Although the elongasome and divisome are spatially segregated during most stages of the growth and cell division cycle of *E. coli*, MreB and PBP2 are recruited to the mid-cell region of dividing *E. coli* at approximately the same time as FtsZ, and they are thought to be required for pre-septal PG synthesis^52–54^. Septal accumulation of PBP2 has also been observed in *Rhodobacter sphaeroides*^55^ and *Hyphomonas neptunium*^56^. At slightly later stages of the division process in *E. coli*, PBP3 is recruited to the septum where it interacts with PBP2^54^. Even though PBP2 accumulates at the septum in *E. coli,* inhibition of its TPase activity does not initially prevent cell division^37^. Our previous two-hybrid studies have shown that PBP2 and PBP3 from *Ct* L2 interact, and we hypothesized that MreB/PBP2 act upstream of PBP3 in chlamydial cell division^18^. The data presented here clearly illustrate that inhibitors of MreB and PBP2 arrest cell division prior to the asymmetric membrane expansion that gives rise to the daughter cell. Although division is also blocked in cells treated with PBP3 inhibitors, asymmetric membrane expansion occurs and division is arrested at an early stage of nascent daughter cell formation. Our results are consistent with pre-septal PG crosslinking by PBP2 being necessary to initiate division and septal PG crosslinking by PBP3 being necessary for the continued growth of the nascent daughter cell in both *Ct* L2 and *C. muridarum*.

To further probe the function of PBP2 and PBP3 during the initiation of the polarized division process in Chlamydiae, we examined the EDA-DA labeled PG structures in *Ct* L2 and *C. muridarum* treated with the PBP-specific antibiotics mecillinam, piperacillin, or penicillin G. In cells treated with mecillinam, which targets PBP2, PG synthesis initiates and it forms a patch just beneath the MOMP-enriched membrane of the cell (Fig. 8B). This PG patch does not mature into a ring when crosslinking of PG by PBP2 is inhibited, and the asymmetric membrane expansion that results in the formation of the nascent daughter cell does not occur. These results suggest the possibility that the PBP2-dependent reorganization of PG into a ring-like structure in *Chlamydia* is required to drive asymmetric membrane growth in the daughter cell. This result is distinct from what has been observed in other bacteria where the absence of PBP2 activity leads to changes in cell shape that indirectly affect cell division.

Treatment of *Ct* L2 and *C. muridarum* with the PBP3 inhibitors, penicillin G and piperacillin, does not prevent the initial enlargement of the nascent daughter cell, and these inhibitors do not prevent the formation of a PG ring at the base of the growing daughter cell in *Ct* L2. However, daughter cell growth in both *Ct* L2 and *C. muridarum* terminates at a discrete early step in polarized division when PBP3 is inhibited. This arrested growth phenotype has not been reported in other bacteria where PBP3 is necessary for the septal PG crosslinking required for fission and new pole synthesis^37^. Our quantitative analyses of the cell division process of *Ct* L2 and *C. muridarum* revealed that relatively few dividing cells have daughter cells that comprise more than 20% of the dividing RB volume. These results suggest that there is a rate limiting step in division, and once dividing cells pass this rate-limiting step they rapidly transition to the two-cell stage. It is possible that this rate-limiting step is the initiation of cross-linking of PG by PBP3. It must be pointed out that all of our conclusions regarding the roles of PBP2 and PBP3 in the chlamydial cell division process are dependent upon the assumption that mecillinam, penicillin G, and piperacillin have similar effects on the enzymatic activities of *E. coli* and chlamydial PBP2 and PBP3.

Although our data point to critical roles for PBP2 and PBP3 in regulating PG organization and polarized division in *Ct* L2 and *C. muridarum*, the mechanisms that drive the polarized assembly of PG in these organisms are not defined. While it is clear that MreB forms rings^15,27^ that overlap the distribution of PG at the septum in dividing *Ct* L2 and that MreB/PBP2 is epistatic to PBP3, previous two-hybrid studies indicated that chlamydial PBP2 does not interact with MreB^18^. This result is consistent with studies in *C. crescentus,* that showed PBP2 does not co-localize with MreB but partially overlaps the distribution of another elongasome component, MreC^57^. The question of how PBP2 and PBP3 and other components of the chlamydial PG biosynthetic machinery are recruited to the MOMP-enriched pole of *Chlamydia* remains unanswered. Importantly, our data have also shown that Hsp60 in *Ct* L2 and *C. muridarum* exhibits a highly ordered reticular pattern of organization that has not been reported in other bacteria. This reticular organization of Hsp60 is unaltered in the presence of a MreB inhibitor. The precise role of MreB and MreC and other potential components of the chlamydial cell division machinery, including RodZ, RodA, FtsK, FtsQ, and FtsW^16,18,24,26,58^, in regulating the highly polarized synthesis of PG in *Chlamydia* and the highly ordered localization of cytosolic proteins such as Hsp60 will be the subject of future investigations.

## Materials and methods

### Antibodies and reagents

Goat polyclonal antibodies directed against the *Chlamydia* major outer membrane protein (MOMP) were purchased from Meridian Life Science. Click-It Labeling Kit for the detection of EDA-DA and various Alexa Fluor conjugated secondary antibodies were purchased from Invitrogen. Affinity purified mouse monoclonal antibodies directed against the *E. coli* RNA polymerase β subunit, which crossreact with the chlamydial RNA polymerase β subunit, were purchased from Biolegend. BODIPY FL C5 ceramide was purchased from Invitrogen. Hoechst 33342 was purchased from ThermoFisher. Penicillin G, piperacillin, d-cycloserine, and mecillinam were purchased from Sigma Chemicals. A22 was purchased from Cayman Chemical.

### Cell culture and *Chlamydia* infections

HeLa cells (ATCC) grown on coverslips in DMEM containing 10% fetal calf serum were infected with *C. trachomatis* serovar L2 (434/Bu strain) or *C. muridarum Nigg* strain (moi = 0.3). At various times post-infection, cells were rinsed with phosphate buffered-saline (PBS) and fixed by incubation with 3% formaldehyde, 0.022% glutaraldehyde in PBS for 2 min then washed in PBS. The cells were permeabilized by incubation in 90% methanol for 1 min, rinsed in PBS, and processed for immunostaining. In some instances, penicillin G (0.2 μM), piperacillin (20 μM), mecillinam (20 μM), d-cycloserine (300 μM), or A22 (100 μM) were added to infected cells for various times prior to fixation.

### Localization analyses

Fixed and permeabilized cells were incubated with primary antibodies and the appropriate Alexa Fluor conjugated secondary antibody prior to imaging on a Zeiss AxioImager. M2 epifluorescent microscope equipped with a 100 × Plan-apochromat oil immersion lens and an AxioCam MRm camera or a Zeiss LSM710 confocal microscope equipped with a 63 × Plan-Apochromat oil immersion lens. Unless otherwise indicated, the images shown in the figures are individual z-slices from a z-stack that extended above and below the cell. In some instances, images were deconvolved using the deconvolution algorithm in the Zeiss Axiovision 4.7 software, and cells were re-oriented in Adobe Photoshop so all cells in a figure were in the same orientation. In addition, 3-dimensional projections of z-stacks were generated using Zeiss Zen Blue software.

### BODIPY-ceramide labeling and live imaging of *Chlamydia*

HeLa cells were infected with *C. trachomatis* serovar L2. At 9.5 hpi 5 µM BODIPY FL C5 ceramide complexed with BSA was added to the media and the infected cells were incubated at 4 °C for 30 min^5,59^. The cells were then washed twice with DMEM and incubated with DMEM/0.7% BSA for one hour to "back-exchange" BODIPY FL C5 ceramide from the plasma membrane. Cells were imaged using a Zeiss LSM710 confocal microscope equipped with a 63 × Plan-Apochromat oil immersion lens. Alternatively, cells were imaged using a 63 × Plan-Apochromat immersible lens on a Zeiss AxioImager. M2 microscope equipped with heatable Universal Mounting Frame and images were collected using an AxioCam MRm camera.

### Quantification of daughter cell and mother cell size in dividing *Chlamydia*

To ensure our analysis was unbiased, every cell undergoing division at various times post-infection was imaged by collecting a z-stack that extended above and below the dividing cell. The largest diameter of the nascent daughter cell and the progenitor mother cell was determined using the measuring tool in the Zeiss AxioVision 4.7 software. This value was used to estimate the volume of the daughter and mother cell according to the formula, v = πr^3^. The boundary between the daughter and mother cell could be defined by increasing the fluorescence intensity of the MOMP staining so the low levels of MOMP in the septum could be visualized. The data in the figures illustrate the percentage the nascent daughter cell and the progenitor mother cell comprise of the total dividing RB. The analysis of cell division intermediates at 18 h post-infection was based on the characterization of dividing cells from eleven inclusions that were imaged in 3 separate experiments.

### PG Labeling in *Ct* L2 and *C. muridarum*

The distribution of PG in dividing *Ct* L2 and *C. muridarum* was determined as described previously^15^. Briefly, HeLa cells were infected with *Ct* L2 or *C. muridarum* and 1 mM ethenyl-modified d-ala- d-ala (EDA-DA) was added to the media at 7.5 hpi or 6 hpi, respectively. *Ct* L2 was fixed at 10.5 hpi and C. muridarum was fixed at 8.5 hpi. The cells were permeabilized as described above. In some experiments, 20 μM mecillinam, 0.2 μM penicillin G, or 20 μM piperacillin was added to infected cells at the same time as EDA-DA. Drug treated cells were fixed at 11.5 hpi (*Ct* L2) or 9.5 hpi (*C. muridarum*) and processed for staining. The EDA-DA, which incorporates into PG, was labeled with Alexa Fluor 594 (red) using the Click-It Labeling Kit from Invitrogen according to manufacturer’s instructions. The cells were then incubated with goat anti-MOMP antibodies followed by donkey anti-goat IgG conjugated to Alexa Fluor 488 (green). After washing, the cells were imaged on an AxioImager.M2 epifluorescent microscope or a Zeiss LSM710 confocal microscope. Images were deconvolved using Zeiss AxioVision 4.7 software.

## Supplementary information

Supplementary Figure Captions.

Supplementary Figure 1.

Supplementary Figure 2.

Supplementary Figure 3.

Supplementary Figure 4.

## References

[1] Gutter B, Asher Y, Cohen Y, Becker Y (1973). Studies on the developmental cycle of Chlamydia trachomatis: Isolation and characterization of the initial bodies. J. Bacteriol..

[2] Nicholson TL, Olinger L, Chong K, Schoolnik G, Stephens RS (2003). Global stage-specific gene regulation during the developmental cycle of *Chlamydia trachomatis*. J. Bacteriol..

[3] Abdelrahman YM, Belland RJ (2005). The chlamydial developmental cycle. FEMS Microbiol. Rev..

[4] Hybiske K, Stephens RS (2007). Mechanisms of host cell exit by the intracellular bacterium Chlamydia. Proc. Natl. Acad. Sci. U. S. A..

[5] Abdelrahman Y, Ouellette SP, Belland RJ, Cox JV (2016). Polarized cell division of *Chlamydia trachomatis*. PLoS Pathog..

[6] Lee KC, Webb RI, Fuerst JA (2009). The cell cycle of the planctomycete *Gemmata obscuriglobus* with respect to cell compartmentalization. BMC Cell Biol..

[7] Fuerst JA (1995). The planctomycetes: Emerging models for microbial ecology, evolution and cell biology. Microbiology.

[8] Santarella-Mellwig R, Pruggnaller S, Roos N, Mattaj IW, Devos DP (2013). Three-dimensional reconstruction of bacteria with a complex endomembrane system. PLoS Biol..

[9] Maxion HK, Liu W, Chang MH, Kelly KA (2004). The infecting dose of *Chlamydia muridarum* modulates the innate immune response and ascending infection. Infect. Immun..

[10] Chen IA (2019). IMG/M v.5.0: An integrated data management and comparative analysis system for microbial genomes and microbiomes. Nucleic Acids Res..

[11] Nguyen BD (2011). Lipooligosaccharide is required for the generation of infectious elementary bodies in *Chlamydia trachomatis*. Proc. Natl. Acad. Sci. U. S. A..

[12] Packiam M, Weinrick B, Jacobs WR, Maurelli AT (2015). Structural characterization of muropeptides from *Chlamydia trachomatis* peptidoglycan by mass spectrometry resolves "chlamydial anomaly". Proc. Natl. Acad. Sci. U. S. A..

[13] Brown WJ, Rockey DD (2000). Identification of an antigen localized to an apparent septum within dividing chlamydiae. Infect. Immun..

[14] Liechti GW (2014). A new metabolic cell-wall labelling method reveals peptidoglycan in *Chlamydia trachomatis*. Nature.

[15] Liechti G (2016). Pathogenic chlamydia lack a classical sacculus but synthesize a narrow, mid-cell peptidoglycan ring, regulated by MreB, for cell division. PLoS Pathog..

[16] Jacquier N, Viollier PH, Greub G (2015). The role of peptidoglycan in chlamydial cell division: Towards resolving the chlamydial anomaly. FEMS Microbiol. Rev..

[17] Matsumoto A, Manire GP (1970). Electron microscopic observations on the fine structure of cell walls of *Chlamydia psittaci*. J. Bacteriol..

[18] Ouellette SP, Karimova G, Subtil A, Ladant D (2012). Chlamydia co-opts the rod shape-determining proteins MreB and Pbp2 for cell division. Mol. Microbiol..

[19] Johnson FW, Hobson D (1977). The effect of penicillin on genital strains of *Chlamydia trachomatis* in tissue culture. J. Antimicrob. Chemother..

[20] Lambden PR, Pickett MA, Clarke IN (2006). The effect of penicillin on *Chlamydia trachomatis* DNA replication. Microbiology.

[21] Wyrick PB (2010). *Chlamydia trachomatis* persistence in vitro: An overview. J. Infect. Dis..

[22] Carballido-Lopez R (2006). Orchestrating bacterial cell morphogenesis. Mol. Microbiol..

[23] Zapun A, Vernet T, Pinho MG (2008). The different shapes of cocci. FEMS Microbiol. Rev..

[24] Stephens RS (1998). Genome sequence of an obligate intracellular pathogen of humans: *Chlamydia trachomatis*. Science.

[25] Gaballah A, Kloeckner A, Otten C, Sahl HG, Henrichfreise B (2011). Functional analysis of the cytoskeleton protein MreB from *Chlamydophila pneumoniae*. PLoS ONE.

[26] Jacquier N, Frandi A, Pillonel T, Viollier PH, Greub G (2014). Cell wall precursors are required to organize the chlamydial division septum. Nat. Commun..

[27] Lee J, Cox JV, Ouellette SP (2020). Critical role for the extended N-terminus of chlamydial MreB in directing its membrane association and potential interaction with divisome proteins. J. Bacteriol..

[28] Moulder JW, Novosel DL, Officer JE (1963). Inhibition of the growth of agents of the psittacosis group by d-cycloserine and its specific reversal by d-alanine. J. Bacteriol..

[29] Tribby II (1970). Cell wall synthesis by *Chlamydia psittaci* growing in L cells. J. Bacteriol..

[30] McCoy AJ, Maurelli AT (2005). Characterization of Chlamydia MurC-Ddl, a fusion protein exhibiting d-alanyl-d-alanine ligase activity involved in peptidoglycan synthesis and d-cycloserine sensitivity. Mol. Microbiol..

[31] Ghuysen JM, Goffin C (1999). Lack of cell wall peptidoglycan versus penicillin sensitivity: New insights into the chlamydial anomaly. Antimicrob. Agents Chemother..

[32] McCoy AJ, Maurelli AT (2006). Building the invisible wall: updating the chlamydial peptidoglycan anomaly. Trends Microbiol.

[33] Skilton RJ (2009). Penicillin induced persistence in *Chlamydia trachomatis*: High quality time lapse video analysis of the developmental cycle. PLoS ONE.

[34] Barbour AG, Amano K, Hackstadt T, Perry L, Caldwell HD (1982). *Chlamydia trachomatis* has penicillin-binding proteins but not detectable muramic acid. J. Bacteriol..

[35] Read TD (2000). Genome sequences of *Chlamydia trachomatis* MoPn and *Chlamydia pneumoniae* AR39. Nucleic Acids Res..

[36] Kocaoglu O, Carlson EE (2015). Profiling of beta-lactam selectivity for penicillin-binding proteins in *Escherichia coli* strain DC2. Antimicrob. Agents Chemother..

[37] Spratt BG (1975). Distinct penicillin binding proteins involved in the division, elongation, and shape of *Escherichia coli* K12. Proc. Natl. Acad. Sci. U. S. A..

[38] Pogliano J, Pogliano K, Weiss DS, Losick R, Beckwith J (1997). Inactivation of FtsI inhibits constriction of the FtsZ cytokinetic ring and delays the assembly of FtsZ rings at potential division sites. Proc. Natl. Acad. Sci. U. S. A..

[39] Lee JK (2018). Replication-dependent size reduction precedes differentiation in *Chlamydia trachomatis*. Nat. Commun..

[40] den Blaauwen T, de Pedro MA, Nguyen-Disteche M, Ayala JA (2008). Morphogenesis of rod-shaped sacculi. FEMS Microbiol. Rev..

[41] Taschner PE, Huls PG, Pas E, Woldringh CL (1988). Division behavior and shape changes in isogenic ftsZ, ftsQ, ftsA, pbpB, and ftsE cell division mutants of *Escherichia coli* during temperature shift experiments. J. Bacteriol..

[42] Nguyen-Disteche M, Fraipont C, Buddelmeijer N, Nanninga N (1998). The structure and function of *Escherichia coli* penicillin-binding protein 3. Cell Mol. Life Sci..

[43] Buddelmeijer N, Beckwith J (2002). Assembly of cell division proteins at the *E. coli* cell center. Curr. Opin. Microbiol..

[44] Weiss DS (1997). Localization of the *Escherichia coli* cell division protein Ftsl (PBP3) to the division site and cell pole. Mol. Microbiol..

[45] Wang L, Khattar MK, Donachie WD, Lutkenhaus J (1998). FtsI and FtsW are localized to the septum in *Escherichia coli*. J. Bacteriol..

[46] Morlot C, Zapun A, Dideberg O, Vernet T (2003). Growth and division of Streptococcus pneumoniae: Localization of the high molecular weight penicillin-binding proteins during the cell cycle. Mol. Microbiol..

[47] Pinho MG, Errington J (2003). Dispersed mode of *Staphylococcus aureus* cell wall synthesis in the absence of the division machinery. Mol. Microbiol..

[48] Wientjes FB, Nanninga N (1991). On the role of the high molecular weight penicillin-binding proteins in the cell cycle of *Escherichia coli*. Res. Microbiol..

[49] Wientjes FB, Nanninga N (1989). Rate and topography of peptidoglycan synthesis during cell division in *Escherichia coli*: Concept of a leading edge. J. Bacteriol..

[50] Curtis NA, Eisenstadt RL, Turner KA, White AJ (1985). Inhibition of penicillin-binding protein 3 of *Escherichia coli* K-12 effects upon growth, viability and outer membrane barrier function. J. Antimicrob. Chemother..

[51] Botta GA, Park JT (1981). Evidence for involvement of penicillin-binding protein 3 in murein synthesis during septation but not during cell elongation. J. Bacteriol..

[52] Den Blaauwen T, Aarsman ME, Vischer NO, Nanninga N (2003). Penicillin-binding protein PBP2 of *Escherichia coli* localizes preferentially in the lateral wall and at mid-cell in comparison with the old cell pole. Mol. Microbiol..

[53] Alexeeva S, Gadella TW, Verheul J, Verhoeven GS, den Blaauwen T (2010). Direct interactions of early and late assembling division proteins in *Escherichia coli* cells resolved by FRET. Mol. Microbiol..

[54] van der Ploeg R (2013). Colocalization and interaction between elongasome and divisome during a preparative cell division phase in *Escherichia coli*. Mol. Microbiol..

[55] Slovak PM, Porter SL, Armitage JP (2006). Differential localization of Mre proteins with PBP2 in *Rhodobacter sphaeroides*. J. Bacteriol..

[56] Cserti E (2017). Dynamics of the peptidoglycan biosynthetic machinery in the stalked budding bacterium *Hyphomonas neptunium*. Mol. Microbiol..

[57] Dye NA, Pincus Z, Theriot JA, Shapiro L, Gitai Z (2005). Two independent spiral structures control cell shape in Caulobacter. Proc. Natl. Acad. Sci. U. S. A..

[58] Ouellette SP, Rueden KJ, AbdelRahman YM, Cox JV, Belland RJ (2015). Identification and partial characterization of potential FtsL and FtsQ homologs of Chlamydia. Front. Microbiol..

[59] Hackstadt T, Scidmore MA, Rockey DD (1995). Lipid metabolism in *Chlamydia trachomatis*-infected cells: directed trafficking of Golgi-derived sphingolipids to the chlamydial inclusion. Proc. Natl. Acad. Sci. U. S. A..

[60] Elwell CA (2011). *Chlamydia trachomatis* co-opts GBF1 and CERT to acquire host sphingomyelin for distinct roles during intracellular development. PLoS Pathog..

[61] Derre I, Swiss R, Agaisse H (2011). The lipid transfer protein CERT interacts with the Chlamydia inclusion protein IncD and participates to ER-Chlamydia inclusion membrane contact sites. PLoS Pathog..

[62] De Benedetti S (2014). Characterization of serine hydroxymethyltransferase GlyA as a potential source of d-alanine in *Chlamydia pneumoniae*. Front. Cell. Infect. Microbiol..

