# Supplementary material for: Penicillin-binding proteins regulate multiple steps in the polarized cell division process of Chlamydia
Source: Sci Rep. 2020 Jul 28;10:12588. doi: 10.1038/s41598-020-69397-x (PMC7387471; doi:10.1038/s41598-020-69397-x)
Supplement: Supplementary file 1 — Supplementary Figure Captions. [file 41598_2020_69397_MOESM1_ESM.docx]

**Supp. Figure 1. Polarized division intermediates of *Ct* L2 detected in fixed and live cells.** HeLa cells infected with *Ct* L2 were fixed at (A) 10.5 hours post-infection. Following permeabilization, cells were incubated with goat anti-MOMP and rabbit anti-Hsp60 antibodies. The cells were then washed with PBS and incubated with donkey anti-goat IgG conjugated to Alexa Fluor 594 (red) and donkey anti-rabbit IgG conjugated to Alexa Fluor 488 (green). Dividing cells were imaged by collecting Z-stacks that extended above and below the cell on a Zeiss AxioImager.M2 microscope. Z-stacks were deconvolved using Zeiss Axiovision 4.7 software. The images shown are representative of dividing cells at this time point. The right-most panel in A is a cell at the two-cell stage of division. (B) Alternatively, HeLa cells infected with *C. trachomatis* serovar L2 were incubated with fluorescent BODIPY FL C5 ceramide as previously described[^5^](#_ENREF_5). This fluorescent lipid analogue is converted to sphingomyelin and incorporated into chlamydial cell membranes[^59-61^](#_ENREF_59). Fluorescently labeled *Chlamydia* undergoing division were imaged in live cells at 11 hours post-infection by collecting Z-stacks on a Zeiss LSM710 confocal microscope. The images were deconvolved using Zeiss Axiovision 4.7 software. The volume of the nascent daughter cell and the progenitor mother cell for each image was calculated as described in the Materials and Methods. The number above each cell in A and B is the percent the daughter cell comprises of the total dividing RB volume. White bars in A and B are 1μM.

**Supp. Figure 2. Quantification of daughter and mother cell volume in dividing *Ct* L2 at 18 hours post-infection.** HeLa cells infected with *C. trachomatis* serovar L2 were fixed at 18 hours post-infection. Following permeabilization, cells were incubated with goat anti-MOMP and rabbit anti-Hsp60 antibodies. The cells were then washed with PBS and incubated with donkey anti-goat IgG conjugated to Alexa Fluor 594 (red) and donkey anti-rabbit IgG conjugated to Alexa Fluor 488 (green). Inclusions were imaged by collecting Z-stacks that extended above and below the inclusion on a Zeiss AxioImager.M2 microscope. Dividing cells were identified and the largest diameter of the nascent daughter and progenitor mother cell in the dividing RBs was determined using the measurement tool in the Zeiss Axiovision 4.7 software. These values were used to estimate the volume of the daughter and the mother cell. The ratio of the volume of the daughter cell (red) and mother cell (blue) to the total volume of the dividing RB is shown in (n=154 cells). A subset of the dividing cells has ratios of daughter and mother cells to total RB volumes that fall between 40 and 60%. We designate these dividing cells as the two-cell stage (green). The daughter and mother cells in this population are similar in size and have a similar content and distribution of MOMP and Hsp60. The change in the number of cells at the two-cell stage between the 10.5 and 18 hour time points is statistically significant (N-1 chi squared: p<0.0001). Each dividing cell that was quantified has both a mother and a daughter cell volume represented in the histogram.

**Supp. Figure 3. Effect of PBP inhibitors on the localization of EF-Tu and the β-subunit of RNA polymerase in *Ct* L2.** HeLa cells were infected with *Ct* L2 and 20μM mecillinam, 20μM piperacillin, or 0.2μM penicillin G was added to the infected cells at 6hpi. The cells were fixed at 11.5hpi and permeabilized. Following washing, cells were incubated with goat anti-MOMP and either mouse antibodies that recognize chlamydial EF-Tu or mouse antibodies that recognize the β-subunit of RNA polymerase. The cells were then washed with PBS and incubated with donkey anti-goat IgG conjugated to Alexa Fluor 594 (red) and donkey anti-mouse IgG conjugated to Alexa Fluor 488 (green). The images were deconvolved using Zeiss Axiovision 4.7 software, and representative images illustrating the effect of the antibiotics on EF-Tu and RNA polymerase localization are shown. White bars are 1μM.

**Supp. Figure 4. Distribution of peptidoglycan during the first division of *C. muridarum* in the absence and presence of PBP inhibitors.** HeLa cells were infected with *C. muridarum* and the peptidoglycan precursor, EDA-DA, was added to infected cells at 6hpi. The cells in panel A were fixed at 8.5hpi and permeabilized. The cells were rinsed and incubated with goat anti-MOMP antibodies while the EDA-DA was labeled with Alexa Fluor 594 (red) using click chemistry technology (Invitrogen). The cells were rinsed and incubated with donkey anti-goat IgG conjugated to Alexa Fluor 488 (green). Cells were imaged by collecting Z-stacks on a a Zeiss AxioImager.M2 microscope. Z-stacks were deconvolved using Zeiss Axiovision 4.7 software. The images shown are representative of dividing cells at this time point. The panel to the right in A is a 3-dimensional projection of the cell marked with the asterisk that was generated using Zeiss Zen Blue software. (B) Alternatively, 20μM mecillinam, 0.2μM penicillin G, or 20μM piperacillin was added to the infected cells at the same time as the EDA-DA. The cells were fixed at 9.5hpi and processed as described above. Collected images were deconvolved using Zeiss Axiovison 4.7 software. The panels on the left are an xy-slice from the collected Z-stack, and the panels on the right are 3-dimensional projections of the Z-stack generated with Zeiss Zen Blue software. White bars in A and B are 1μM.
