# Supplementary figures and images for: Penicillin-binding proteins regulate multiple steps in the polarized cell division process of Chlamydia
Source: Sci Rep. 2020 Jul 28;10:12588. doi: 10.1038/s41598-020-69397-x (PMC7387471; doi:10.1038/s41598-020-69397-x)

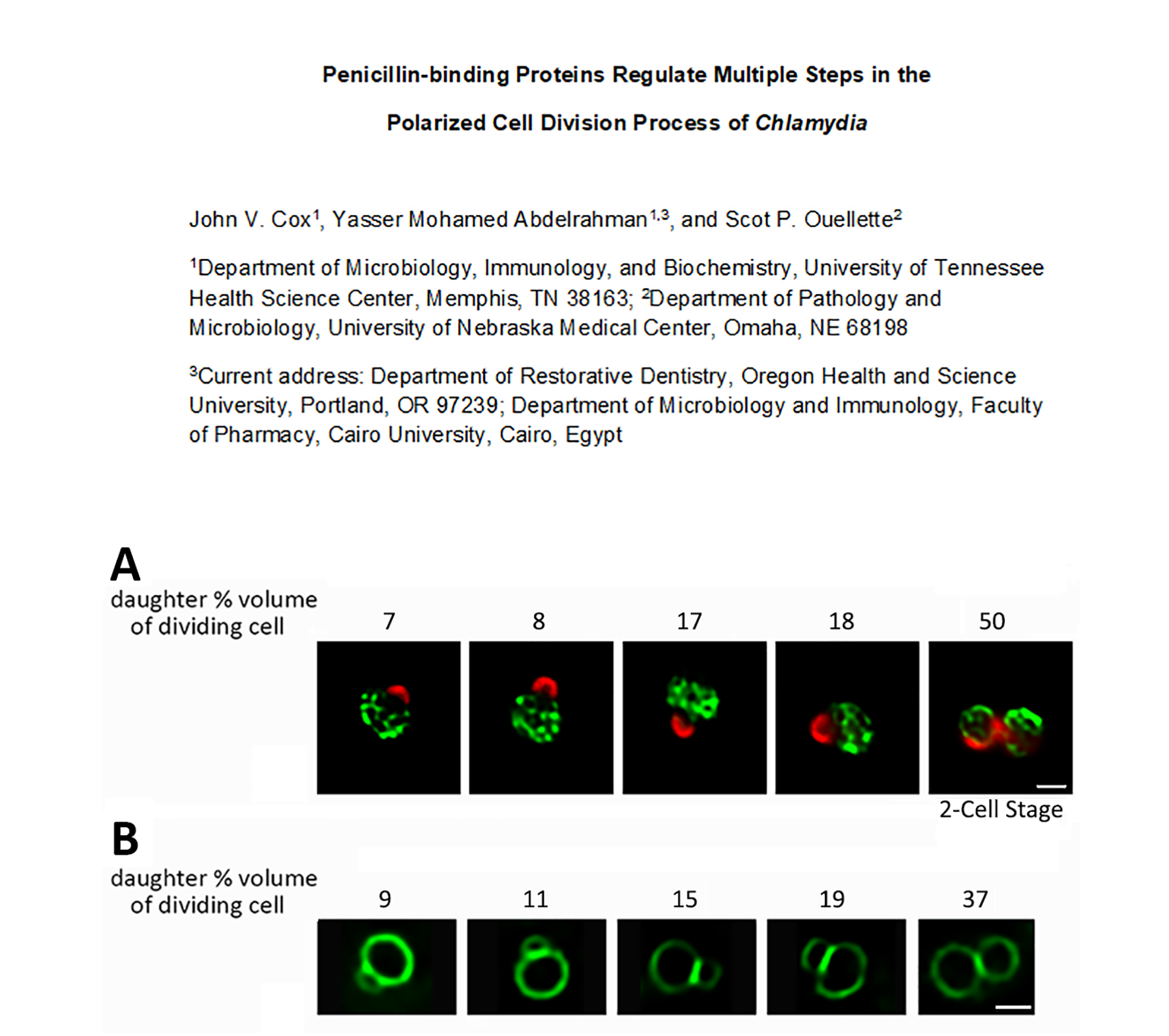

Supplement: Supplementary file 2 — Supplementary Figure 1. [file 41598_2020_69397_MOESM2_ESM.tif]

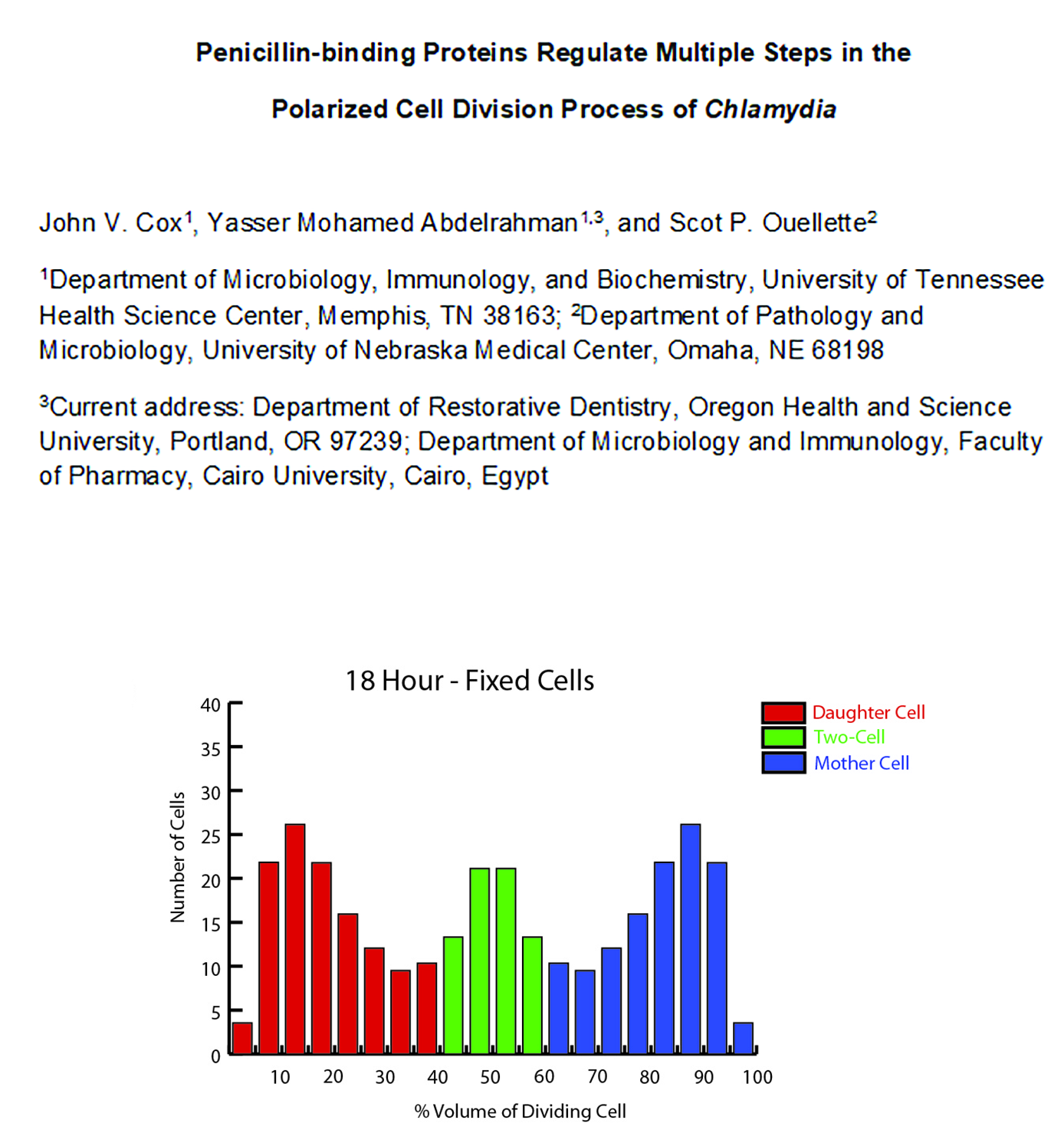

Supplement: Supplementary file 3 — Supplementary Figure 2. [file 41598_2020_69397_MOESM3_ESM.tif]

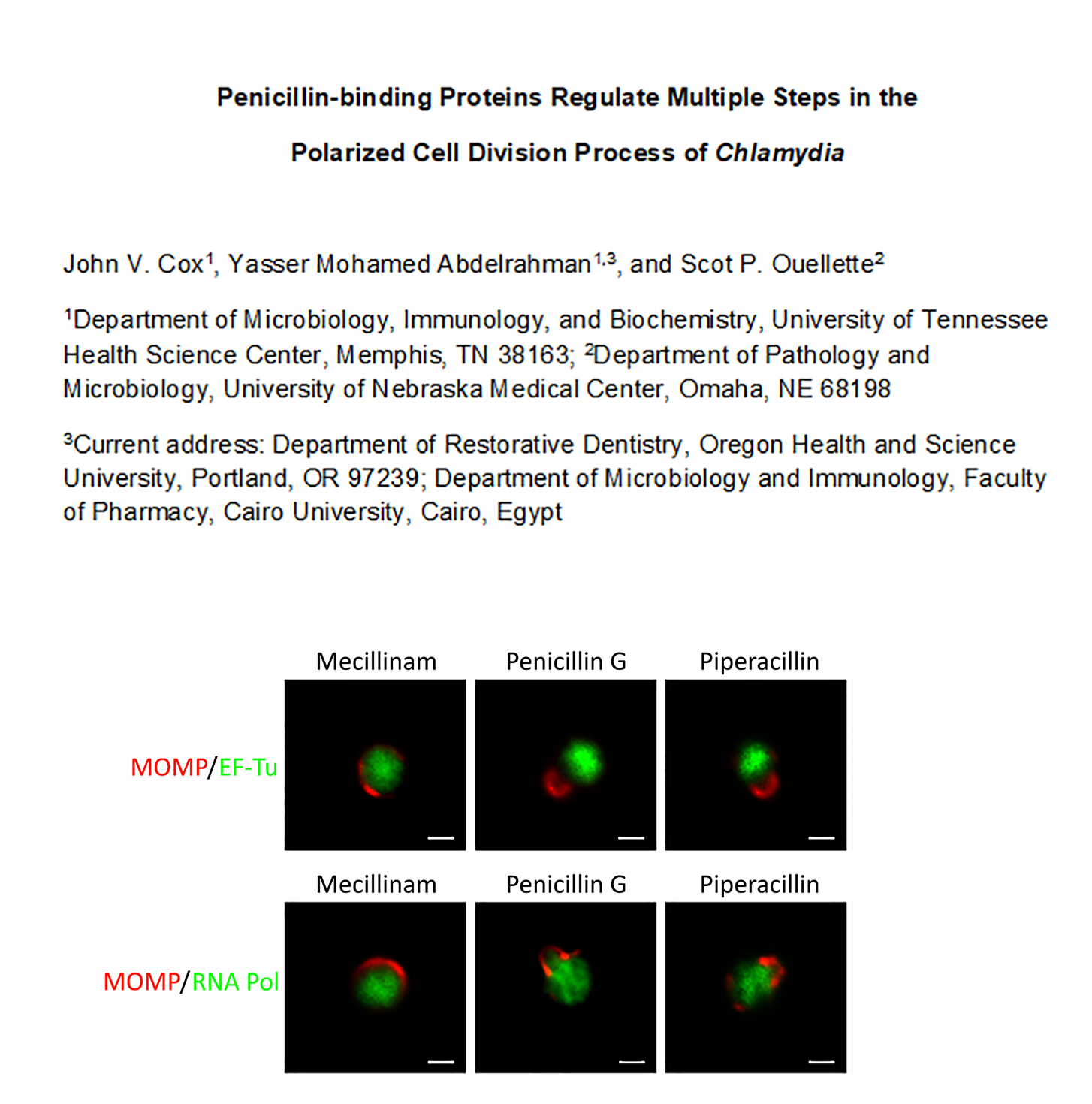

Supplement: Supplementary file 4 — Supplementary Figure 3. [file 41598_2020_69397_MOESM4_ESM.tif]

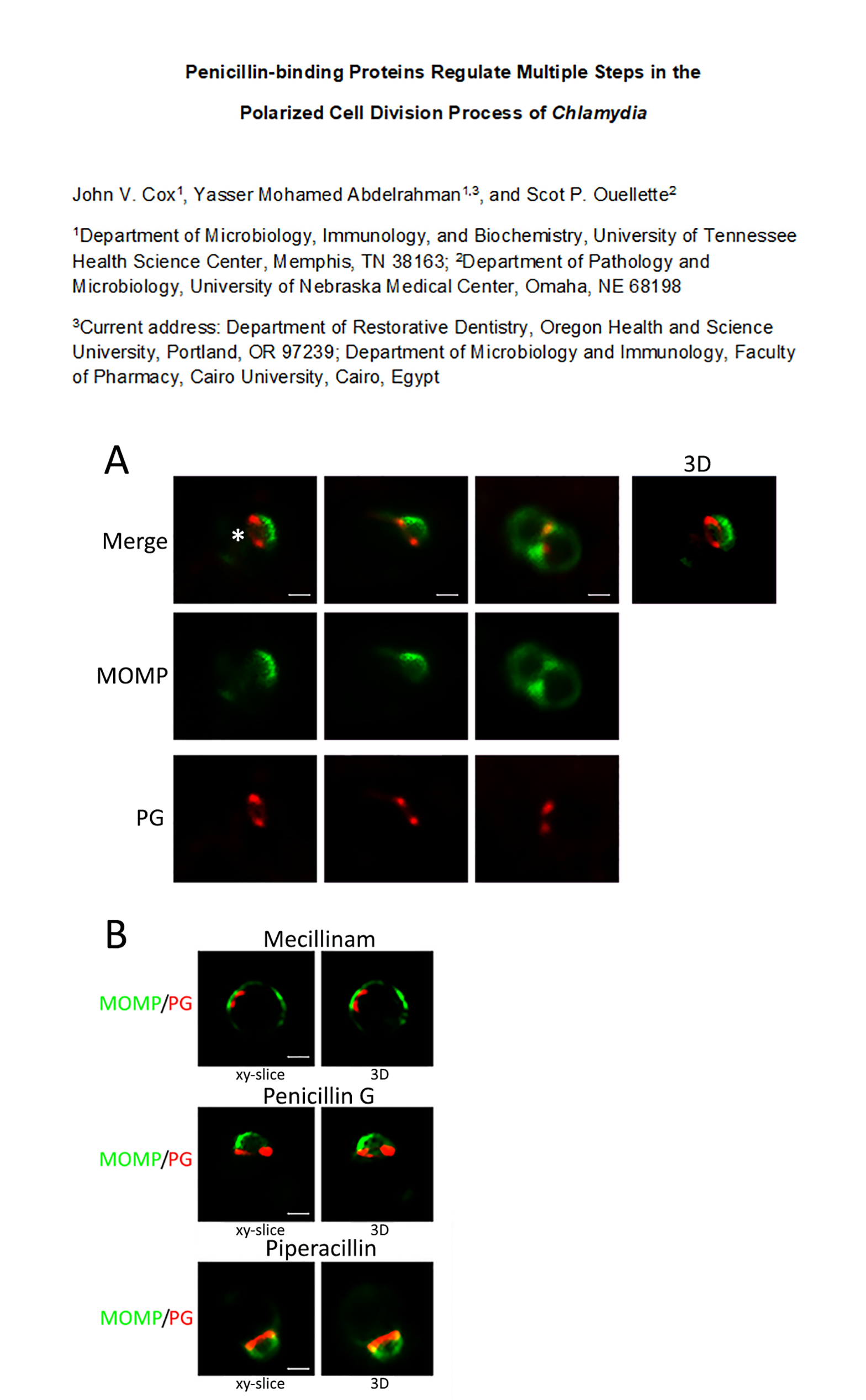

Supplement: Supplementary file 5 — Supplementary Figure 4. [file 41598_2020_69397_MOESM5_ESM.tif]
